# IoT smartwatch based on open technologies for the collection of thermal comfort data

**DOI:** 10.1016/j.ohx.2025.e00633

**Published:** 2025-03-03

**Authors:** Julio Landa, Guillermo Barrios, Guadalupe Huelsz

**Affiliations:** Instituto de Energías Renovables, Universidad Nacional Autónoma de Mexico, Priv. Xochicalco S/N Temixco Morelos, CP 62580, Mexico

**Keywords:** Thermal comfort, IoT, Smartwatch, Physiological measurements, Open-source hardware

## Abstract

This paper presents an IoT smartwatch based on open technologies for thermal comfort data collection. The device performs simplified thermal comfort surveys every hour, collecting data such as clothing insulation level (clo), metabolic activity (met), location, thermal sensation, and thermal acceptance. In addition, it measures two physiological variables: skin temperature and heart rate. The smartwatch is built with low-cost components, including a XIAO ESP32C3 microcontroller, a GY-906 temperature sensor, a MAX30102 heart rate sensor, and Seeed Studio’s XIAO Round Display touchscreen. All collected data are published in real time on an IoT platform that allows remote access to all information. During device registration, the user is prompted to complete a Google Form, where additional data such as gender, age, height, weight, and frequency of air conditioner use are collected. This information, in combination with data from the smartwatch, contributes to a robust database. The ease of use, the design of the device, and its open-source nature make it ideal for the collection of thermal comfort data and its potential use for the generation of adaptative thermal comfort models.

## Graphical abstract

**Figure:**
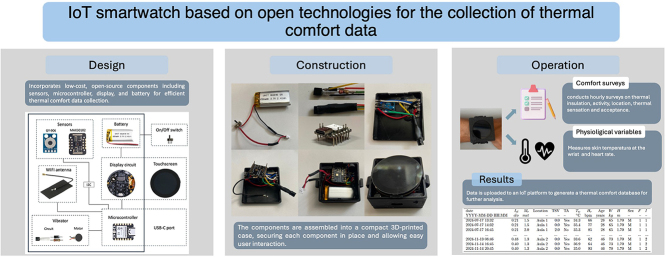

## Specifications table

**Table:** 

Hardware name	IoT Smartwatch for thermal comfort data.

Subject area	• Engineering • Environmental • Educational tools and open source alternatives to existing infrastructure

Hardware type	• Collecting thermal comfort surveys and measuring physiological variables

Closest commercial analog	fitbit + cozie, apple watch + co2

Open source license	GNU General Public License version 3 (GPLv3)

Cost of hardware	$49 USD

Source file repository	https://doi.org/10.5281/zenodo.14835096

## 1 Hardware in context

The study of thermal comfort and energy efficiency in buildings has become increasingly important in recent years. Bioclimatic design, which adapts building design according to the local climate [1], has emerged as a fundamental approach in thermal comfort and energy efficiency. Concurrently, the rapid advancement of digitalization, including the Internet of Things (IoT), has enabled the application of new technologies in data measurement, collection of data, and automatization [2]. The development of adaptive thermal comfort models to be used in the bioclimatic design process and energy efficiency for buildings can be carried out only through surveys of thermal comfort perception, measurements, and collect data. Depending on the type of adaptive comfort model, the measurements can be only on air temperature, as suggested by Humphreys and Nicol [3], or on a wider range of environmental and human related thermal variables, as the PMVe model by Fanger and Toftum [4] proposed.

In pursuit of a deeper understanding of thermal comfort, efforts have been undertaken to link physiological variables with thermal comfort perception. In a literature review, several studies have found that wrist skin temperature is considered a clear indicator of thermal comfort or discomfort [5], as it can discern between neutral thermal sensations and those that are slightly cool or warm [6], [7]. Other studies have highlighted that the most commonly used variables to be related to thermal comfort were skin temperature in different areas and heart rate [7], [8]. A strong correlation has been found between thermal comfort sensations and skin temperature measurements in various parts of the body, including the upper wrist, radial artery, ulnar artery, and index fingertip [7]. Similarly, a subsequent study recorded more physiological variables and confirmed the strong correlation between thermal comfort and skin temperature on the wrist [9].

Heart rate variability measured with the commercial sensor myBeat has been used to train a machine learning algorithm to model personal thermal comfort [10]. Core temperature and thermal comfort are predicted with the use of a Fitbit with the integration of iButton [11] measuring proximity air temperature, humidity, heart rate, skin temperature, skin humidity, and thermal sensation vote (TSV) [11]. Thermal comfort surveys have been collected using the framework Cozie for smartwatches, which is available for Apple and was available for selected Fitbit devices [12]. Personalized comfort models have been developed with thermal comfort surveys in home, office, or in transitions from place to place together with physiological parameters, personal information such as sex, age and body mass index, and indoor or outdoor air temperature using iButton [13]. A fully open ecosystem that seamlessly integrates both comfort surveys and physiological measurements within a single wearable device remains lacking in the field.

Our project involves the development of a smartwatch that performs periodic thermal comfort surveys and measurements of wrist skin temperature and heart rate. The smartwatch collects data and sends it to an IoT platform, allowing real-time access to all information. A simplified thermal comfort survey gathers data on the level of thermal isolation of clothes, the activity performed during the survey, thermal sensation vote, and thermal acceptance. The smartwatch prompts the user to take the survey every hour. The smartwatch sets the base to create a fully open ecosystem integrating comfort surveys and physiological measurements.

## 2 Hardware description

**Fig. 1:**
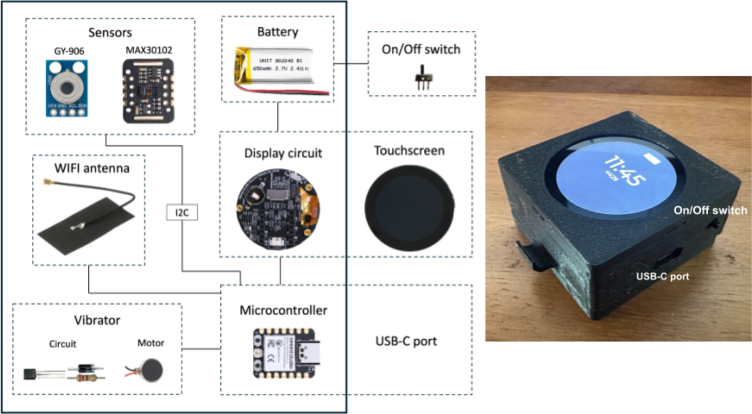
Block diagram (left) and the smartwatch (right). The solid box in the diagram encloses the elements inside the case and the excluded elements are those that provide interaction with the user. The solid lines indicate physical connections between elements.

The smartwatch consists of the XIAO round display and a XIAO ESP32C3 microcontroller. For heart rate measurement, the MAX30102 pulse sensor is used, while wrist skin temperature is measured with the GY-906 infrared temperature sensor. Alerts for the user to complete thermal comfort surveys are provided through a vibrating motor. A microswitch allows the user to turn the smartwatch on and off. The smartwatch is charged via USB-C and it uses a LiPo battery. A block diagram of the parts for the smartwatch is shown in Fig. 1 and the finished smartwatch at the right.

The XIAO ESP32C3 by Seeed Studio features a 32-bit RISC-V processor at 160 MHz, integrated with 2.4 GHz Wi-Fi (with an antenna) and Bluetooth 5.0. This compact board measures just 21.0 mm × 17.5 mm and includes UART, I2C, and SPI interfaces, along with 400 kB SRAM and 4 MB flash memory. The Seeed Studio Round Display for XIAO features a 39 mm diameter capacitive round touchscreen with a 240 × 240 resolution capable of displaying 65 K colors. It is compatible with all XIAO series boards and includes a built-in RTC, a battery charge circuit, and a microSD slot supporting up to 32 GB. The GY-906, also known as the MLX90614, is an infrared thermometer designed for non-contact temperature measurements from −70 °C to 380 °C with an accuracy of ±0.5 °C and a resolution of 0.02 °C. It uses I2C communication and operates at 3.7 V. The MAX30102 is an integrated pulse oximetry and heart rate monitoring sensor module based on photoplethysmography. It features two LEDs, a photodetector, and low-noise electronics, supporting I2C communication. It operates from 3.3 V to 5.0 V. A 3.7 V LiPo battery with a capacity of 650 mAh provides an autonomy of approximately 8 h.

The key features of the present smartwatch are:

• Simple and low-cost design utilizing XIAO ESP boards and a XIAO touchscreen display, eliminating the need for a custom PCB.
• Adaptable design featuring the I2C sensors, MAX30102 and GY-906, allowing for easy replacement or the addition of new sensors with minimal modification.
• Vibrating alarm system that prompts the user to respond to a survey for additional data collection.
• Simplified thermal comfort survey interface programmed in open-source library LVGL, allowing surveys to be developed from scratch for other purposes using a touchscreen interface.
• Data collected by an IoT platform.

## 3 Design files summary

Table 1 presents the FreeCAD files to export the STL files to print the case, the schematic, and the firmware files responsible for the operation of the sensors, the thermal comfort survey, and the publication of data to the IoT platform. The file names are in Spanish because we want to facilitate the adoption of the project in the Spanish-speaking community.

**Parte_inferior**: the bottom part of the case, in contact with the wrist, it incorporates appropriate supports to secure the sensors in position.

**Table 1:** Design files for the smartwatch.

Design filename	File type	Open source license	Location of the file
Parte_inferior.FCStd	CAD	CERN OHl	/Carcasa/
Parte_central.FCStd	CAD	CERN OHl	/Carcasa/
Parte_superior.FCStd	CAD	CERN OHl	/Carcasa/
Seguro.FCStd	CAD	CERN OHl	/Carcasa/
Diagrama_conexiones.fzz	Schematic	CERN OHl	/Esquematicos
Proyecto_confort.ino	Arduino sketch	GNU GPL	/Proyecto_confort/
ui_Inicio.c	C++	GNU GPL	/Proyecto_confort/
ui_Clo.c	C++	GNU GPL	/Proyecto_confort/
ui_Met.c	C++	GNU GPL	/Proyecto_confort/
ui_Ubicacion.c	C++	GNU GPL	/Proyecto_confort/
ui_Sensacion.c	C++	GNU GPL	/Proyecto_confort/
ui_Aceptacion.c	C++	GNU GPL	/Proyecto_confort/
ui.c	C++	GNU GPL	/Proyecto_confort/
ui.h	Header file	GNU GPL	/Proyecto_confort/
ui_helpers.c	C++	GNU GPL	/Proyecto_confort/
ui_helpers.h	Header file	GNU GPL	/Proyecto_confort/
ui_events.c	C++	GNU GPL	/Proyecto_confort/
ui_events.h	Header file	GNU GPL	/Proyecto_confort/

**Parte_central**: the middle part of the case, where the microcontroller, the vibrating motor, the screen, and the On/Off switch are placed and the straps are hooked.

**Parte_superior**: the top part of the case, it holds the touchscreen in place.

**Seguro**: holds the switch in place.

**Diagrama_conexiones**: wiring diagram of the microcontroller, the sensors, and the vibrating motor circuit.

**Proyecto_confort.ino**: the main Arduino sketch file.

**ui_Inicio.c**: the home screen of the smartwatch interface.

**ui_Clo.c**: screen with checkboxes to calculate clo.

**ui_Met.c**: screen to select activity to determine met.

**ui_Ubicacion.c**: screen to select location inside a building.

**ui_Sensacion.c**: screen to cast the thermal sensation vote.

**ui_Aceptacion.c**: screen to accept or decline the thermal sensation.

**ui.c**: main file where all screens for the thermal comfort survey are gathered.

**ui.h**: header file where the functions to be used in ui.c are declared.

**ui_helpers.c**: provides helper functions for the User Interface (UI) components.

**ui_helpers.h**: declares helper functions for UI components.

**ui_events.c**: handles event callbacks for UI interactions and navigation.

**ui_events.h**: declares event callback functions for UI interactions and navigation.

## 4 Bill of materials summary

In Table 2, the bill of materials for this project is presented. Additionally, soldering, silicone or glue, and adhesive tape may be required.

**Table 2:** Bill of materials for the smartwatch.

Designator	Component	Number	Unit cost	Total cost	Source of materials
			(USD)	(USD)	
MCU	XIAO ESP32C3 microcontroller unit with Wi-FI antenna	1	7.49	7.49	Seeed Studio
battery	650 mAh LiPo battery	1	2.5	2.5	UNIT electronics
switch	2.54 mm pitch switch	1	0.12	0.12	UNIT electronics
battery connector	JST 1.25 mm male connector	1	0.35	0.35	Amazon
transistor	2n2222 transistor	1	0.06	0.06	UNIT electronics
resistor	220 Ω resistor	1	0.02	0.02	UNIT electronics
diode	1N4007 diode	1	0.06	0.06	UNIT electronics
vibrator	Vibrator motor	1	0.76	0.76	UNIT electronics
termofit	10 mm termofit	1	0.1	0.1	UNIT electronics
MAX30102	MAX30102 pulse sensor	1	1.93	1.93	UNIT electronics
GY-906	GY-906 infrared temperature sensor	1	16.05	16.05	UNIT electronics
headers	2.54 mm female header	1	0.22	0.22	UNIT electronics
straps	Cable ties	2	0.11	0.22	Amazon
display	XIAO round display	1	18	18	Seeed Studio
cables	22 AWG cable	0.5 m	0.24	0.12	UNIT electronics
case	PLA filament	50 g	0.02	1	UNIT electronics

## 5 Build instructions

### 5.1 Building instructions

**Fig. 2:**
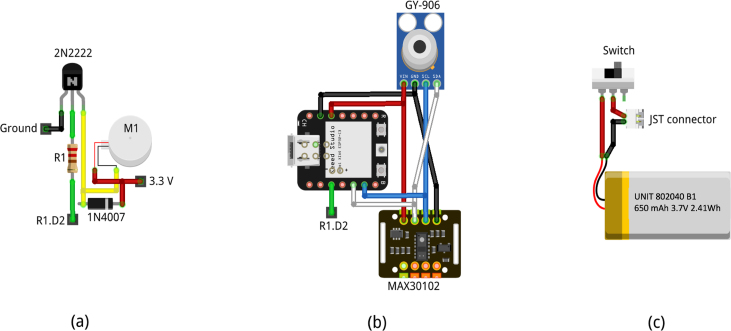
Schematics for (a) vibrating motor for alarm circuit, (b) sensors and MCU circuit, and (c) battery circuit.

To build the smartwatch, it is essential to have the schematics presented in Fig. 2 readily available. The electronics are divided into three main circuits: the vibrating motor for alarms circuit (Fig. 2(a)), the sensors and MCU circuit (Fig. 2(b)), and the battery circuit (Fig. 2(c)). The color code used in the schematic is:

• **Red**: Vcc connection (3.3 V).
• **Black**: Ground.
• **Blue**: communication cable SCL.
• **White**: communication cable SDA.
• **Green**: motor activation signal.
• **Yellow**: connection between transistor emitter pin and vibrating motor.

Once familiarized with the schematic, the case must be printed, the sensors prepared, the vibrating circuit constructed, the MCU prepared, and the battery circuit assembled. These components are then mounted into the bottom part of the case, the MCU is connected, the middle part of the case is placed together with the battery circuit, followed by the display, and finally the top part of the case and the straps. Each step is detailed below.

**Fig. 3:**
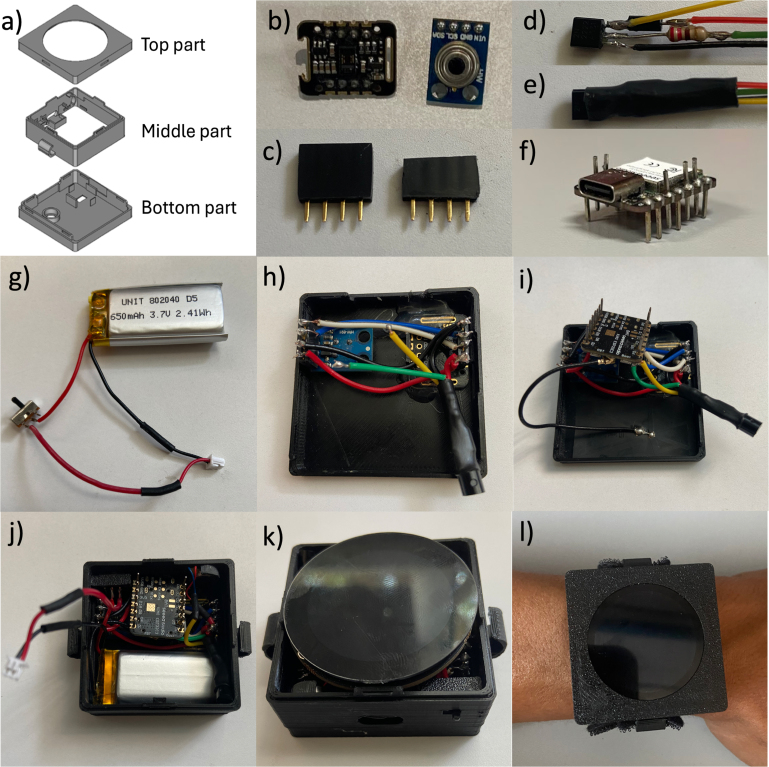
Photos of the building instructions. (For interpretation of the references to color in this figure legend, the reader is referred to the web version of this article.)

To print the case, the STL files must be exported from the FCStd files. The case comprises four parts: bottom, middle, latch, and top. The bottom part of the case has two holes to hold the sensors. The middle part of the case holds the switch, the vibrating motor, and the MCU. The latch secures the switch at the middle part of the case. The top part of the case secures the screen. The bottom, middle and top parts of the case are designed to snap-fit together and are shown in Fig. 3(a). It is recommended to configure a layer height of 0.1 mm or an even higher quality configuration for best results for the 3D printing.

The next step is to prepare the sensors with headers to facilitate replacement if needed. Male headers are soldered to both sensors, but the plastic insulation from the male headers is removed and the pins are cut to the same length as the removed plastic (2.54 mm) to reduce size (Fig. 3(b)). Female headers connect the sensors to the MCU to provide a plug-and-play design. The plastic base of the female headers is also cut by about 2.54 mm to reduce dimensions, as shown in Fig. 3(c). Unless otherwise specified, all following connections are soldered to the headers’ pins.

To build the vibrating circuit, follow the schematic in Fig. 2(a), connect the transistor, resistor, and diode (Fig. 3(d)). The wiring should have a minimum length of 5 cm and fit the circuit inside the termofit, which should looks like Fig. 3(e). Do not connect the motor yet.

To prepare the MCU, solder the header pins to the board, ensuring that specific pins protrude from the top side for easy access. These pins are power supply pins (Ground, 3.3 V), I2C communications pins (SDA, SCL), and digital pin (D2) (Fig. 3(f)). Remove the plastic insulation from the headers and cut the length of the MCU pins so that they can be positioned directly above the display.

Subsequently connect the battery, switch, and JST connector according to the diagram in Fig. 2(c). The ground (black) wire should be 4.5 cm long. Two segments are needed for the 3.7 V wire (red): one from the JST connector to the switch (7 cm) and another from the switch to the battery (3.5 cm), as shown in Fig. 3(g).

Once these steps are completed, the assembly proceeds. Plug the female headers to each sensor and fit the sensors into the bottom part of the case, ensuring the headers are aligned and flush against the wall of the case. Secure the sensors with silicon or adhesive tape. Solder the power pins of the vibrating circuit to the power pins of the MAX30102 following Fig. 2(b). The yellow and green wires from the vibrating circuit are not connected yet (Fig. 3(h)). Dupont wire is used, but 22 AWG or a heavier gauge wire can also be used to facilitate connections and space management inside the smartwatch.

The MCU is placed upside down and wired to the sensors following the diagram in Fig. 2(b). The power pins are connected to the GY-906 sensor, while the I2C pins are connected to the MAX30102 sensor. Connect the MCU’s digital pin to the vibrator circuit (R1.D2). Connect the antenna to the MCU and fix it on the bottom part of the case in the free space next to the sensors. The connections should look like those shown in Fig. 3(i).

The next step is to assemble the components into the middle part of the case. Align the USB-C port from the MCU with its position on the middle part of the case. Place the switch and secure it with the latch. Insert the motor into the round compartment on the middle part of the case and solder its pins according to the diagram in Fig. 2(a). Assemble the middle and bottom parts of the case, and position the battery in the space next to the MCU and above the Wi-Fi antenna. Place the vibrator circuit in the corner next to the battery, as shown in Fig. 3(j).

Then, connect the screen to the battery using the JST connector and mount the screen onto the MCU, as shown in Fig. 3(k).

Finally, attach the top part of the case and the straps on the sides carefully to avoid damaging the case, as shown in Fig. 3(l). The bottom, middle and top parts of the case interlock with a clicking sound, ensuring a secure lock.

### 5.2 Configuration

1. Install Arduino IDE
  • Download and install the latest version of the Arduino IDE from its official website [14].
2. Add ESP32 board package to Arduino IDE
  • Open the Arduino IDE and click on “File” followed by “Preferences”.
  • In the “Additional Boards Manager URLs” box, paste the URL: https://raw.githubusercontent.com/espressif/arduino-esp32/gh-pages/package_esp32_index.json.
  • Navigate to “Tools”, select “Board”, and then “Boards Manager”. Search for “esp32” and install the esp32 Expressiff System.
3. Set up project environment
  • From the repository at https://doi.org/10.5281/zenodo.14835096, copy all files from the Arduino folder into the existing Arduino folder in your Documents directory.
4. Register device on Thingsboard
  • Create a new device on Thingsboard (or an alternative IoT platform).
  • Complete the Google Form with device and user information. This form collects information about the user of the SmartWatch such as age, sex, weight, height, frequency of use of air conditioning spaces, etc.
5. Configure and upload code
  • Download Proyecto_confort folder and in the in Proyecto_confort.ino file navigate to configure the Wi-Fi network by setting the SSID and password:

**Figure:**
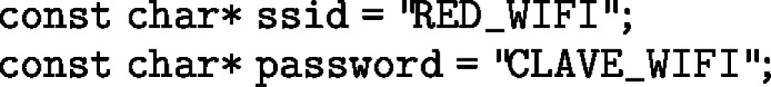
  • Set up the Thingsboard token device and server address with the following lines in the same file:

**Figure:**
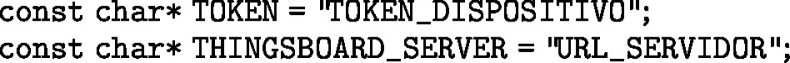
  • Connect the MCU to your computer, compile the code in the Arduino IDE, and upload it to the device selecting XIAO_ESP32C3 board.

The smartwatch retrieves the current time from the internet connection and publishes the data an installed copy of the Community Edition of ThingsBoard, an open-source platform with comprehensive documentation on installation, data publishing, and collection [15]. The Arduino code can be adapted to use ThingSpeak [16] or any other IoT platform.

## 6 Operation instructions

Once the smartwatch is assembled, charge it using the USB-C port. Ensure the power switch is turned on to allow the device to charge. Turn on the smartwatch, adjust the straps to ensure it stays secure during measurements, and wait for the smartwatch to prompt you to complete the on-device survey.

**Fig. 4:**
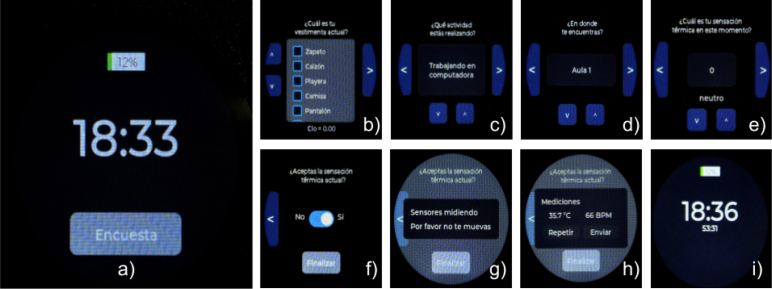
Screens from the survey: (a) Start survey, (b) clothing selection (clo level) with total clo at bottom of the screen, (c) activity selection, (d) location selection, (e) thermal sensation vote, (f) thermal acceptance, (g) skin temperature and heart rate measurement in progress screen, (h) results and option to measure again or send the data, (i) main screen with timer for the next survey. All screens are in spanish.

When the vibrating alarm activates, the user can choose to start the survey at any moment (Fig. 4a). The first step requires the user to select the clothing they are wearing (Fig. 4b). The selected clothing is saved, and the total clo value is displayed on the screen. Next, the user is prompted to select their activity from a predefined list to determine the metabolic rate (Fig. 4c). Following this, the user must choose their location from another predefined list (Fig. 4d).

The survey then requests a thermal sensation vote, with values ranging from −3 to 3 in 0.5 increments (Fig. 4e). The user is also asked to specify whether they find the thermal sensation acceptable (Fig. 4f). Subsequently, a screen appears while the sensors measure wrist skin temperature and heart rate (Fig. 4g). Before publishing the results to the IoT platform, the smartwatch displays the measured wrist skin temperature and heart rate, asking the user whether the measurements should be repeated (Fig. 4h). The user can either repeat the measurement or complete the process.

The options available in the predefined lists for clothing, activity, and location can be modified by editing the corresponding files in the smartwatch’s code. Specifically, the code for the clothing selection screen is located in the file ui_Clo, the activity selection in ui_Met, and the location selection in ui_Ubicacion. Similarly, the thermal sensation vote, based on the scale from −3 to 3 in increments of 0.5 [17], is defined in the file ui_Sensacion, while the thermal comfort acceptability question is implemented in ui_Aceptacion. After completing all the questions, the smartwatch retains the user’s responses as defaults for the next survey. These defaults allow the user to make quick adjustments or proceed without re-entering all data.

The smartwatch and the registration form collect the data presented in Table 3. All variables originating from sensors or surveys are published in the IoT platform each time the smartwatch is used, whereas the variables collected during the registration process are considered constant throughout the period the user utilizes the smartwatch. To retrieve the measurements, a Python Jupyter notebook was designed. This notebook download all data from each survey and measurement performed and gathers all information from the smartwatch and the registration form into a dataframe. The notebook is located in the repository at the folder Thingsboard.

**Table 3:** Variables collected from the smartwatch and registration form.

Variable	Units	Origin	Sensor/Survey
Heart rate	[bpm]	Smartwatch	MAX30102 pulse sensor
Wrist skin temperature	[°C]	Smartwatch	GY-906 sensor
Clothing insulation level	[clo]	Survey	Predefined clo values
Metabolic rate	[met]	Survey	Predefined met values
Location	[–]	Survey	Predefined locations
Thermal sensation vote	[–]	Survey	Scale from −3 to 3 (0.5 steps)
Thermal acceptance	[–]	Survey	Yes/No
Age	[year]	Registration	
Weight	[kg]	Registration	
Height	[m]	Registration	
Sex	[–]	Registration	
Frequency of air-conditioned use	[–]	Registration	
User ID	[–]	Registration	

## 7 Validation and characterization

Section 7.1 presents the calibration for the skin temperature sensor and Section 7.2 presents the calibration for the heart rate sensor. Section 7.3 shows the validation of the smartwatch to collect data from sensors and the survey.

### 7.1 GY-906 calibration

The GY-906 sensor was calibrated using the AET-R1B1 infrared body thermometer with 0.2 °C accuracy and 0.1 °C resolution. The emissivity was set to 0.98 in the GY-906 sensor, which corresponds to human skin emissivity [18], [19]. Experiments were conducted by directly comparing measurements from the GY-906 sensor and the AET-R1B1 infrared thermometer. Skin temperature measurements were taken on the posterior part of the wrist, collected over a span of three days. This resulted in a total of 115 pairs of measurements. The data statistics are summarized in Table 4.

The Mean Error (ME) and the Mean Absolute Error (MAE) were used for calibration. The ME is defined as

(1) $$\text{ME}=\frac{1}{n}\overset{n}{\underset{i=1}{\sum }}\left(X_{s,i}-X_{r,i}\right),$$

where $X_{s,i}$ and $X_{r,i}$ are the sensor and the reference measurements, respectively, for the ith observation, and n is the number of paired measurements. The ME quantifies the mean deviation of the sensor from the reference. A positive value indicates that the sensor readings are systematically higher than the reference, while a negative ME indicates the opposite. The MAE is defined as

(2) $$\text{MAE}=\frac{1}{n}\overset{n}{\underset{i=1}{\sum }}\left|X_{s,i}-X_{r,i}\right|,$$

which quantifies the average magnitude of the deviations between the sensor and the reference measurements. Because MAE considers only absolute differences, it provides a clear measure of the overall accuracy of the sensor.

**Table 4:** Statistics of AET-R1B1 infrared body thermometer and GY-906 measurements.

Statistics	AET-R1B1 [°C]	GY-906 [°C]
Mean	36.7	34.6
Standard deviation	0.7	1.0
Minimum	32.8	30.6
Maximum	38.6	36.9

**Fig. 5:**
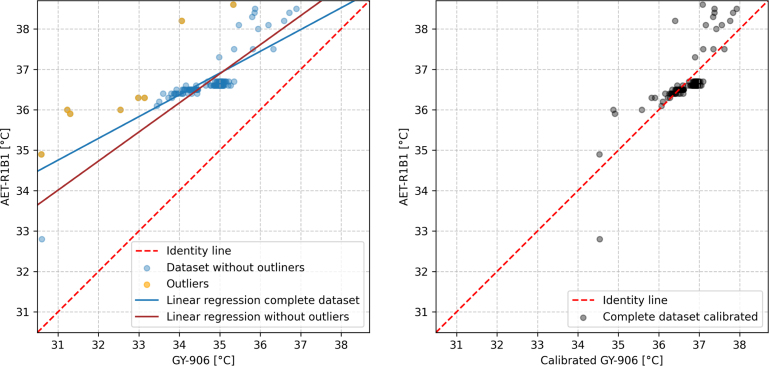
(a) Scatter plot comparing the GY-906 sensor measurements with AET-R1B1 infrared body thermometer. Blue points represent the dataset without outliers, orange points are the outliers, solid blue line is the linear regression for the complete dataset, red solid line is the linear regression for the dataset without outliers and the dashed line is the identity line, (b) the complete dataset calibrated (gray points) and the identity line. (For interpretation of the references to color in this figure legend, the reader is referred to the web version of this article.)

The analysis for the dataset with 115 measurement pairs consisted in a Z-score test with a criterion of 1.5 that revealed eight outliers, which were subsequently identified and removed. To assess the impact of these outliers, we performed a linear regression on both the original dataset and the outlier-excluded dataset. For the original dataset, the best-fit equation is given by $T_{r}=0.54T_{s}+18.06\;$°C whereas the regression for the outlier-excluded subset yields $T_{r}=0.72T_{s}+11.71\;$°C, where $T_{r}$ and $T_{s}$ are the temperature corresponding to the reference device (AET-R1B1) and the sensor (GY-906). Next, we calculated the ME and the MAE for both datasets with the linear regression applied. In the original set, the ME = −3.12 × $10^{-15}\;$°C and the MAE = 0.3 °C. For the dataset without outliers the ME = −2.9 × $10^{-15}\;$°C and the MAE = 0.3 °C. Fig. 5(a) illustrates the data without outliers (blue), the identified outliers (orange), and linear regression lines for the complete dataset and the dataset without outliers. Fig. 5(b) shows the calibrated measurements for the complete dataset alongside the identity line. Since both datasets yielded the same mean error and mean absolute error, it was opted to use the linear regression of the complete dataset.

### 7.2 MAX30102 calibration

The Yonker YK-81C oximeter was used as a reference to calibrate the heart rate sensor. This oximeter has an accuracy of ±1 beats per minute (bpm). Simultaneous heart rate measurements were conducted using both the MAX30102 and the oximeter, with readings taken at 10-s intervals. At each interval, the corresponding oximeter reading was recorded. Initial measurements from the MAX30102, under default settings, showed a MAE of 18.5 bpm. The default settings are [20]:

• RATE_SIZE = 4,
• sampleAverage = 4,
• ledBrightness = 31,
• ledMode = 2,
• sampleRate = 400,
• pulseWidth = 411,
• adcRange = 2048,

To balance accuracy and total measurement time, only intermediate values of RATE_SIZE (4, 6, and 8) were explored using the proposed 10-s measurement intervals. Table 5 shows the MAE of the heart rate for different values of RATE_SIZE, the best value for RATE_SIZE was 6 with a MAE of 4.4 bpm.

Subsequently RATE_SIZE was kept at 6 and sampleAverage was varied from 4 to 8. The best MAE was 3.6 bpm with sampleAverage = 8. Thus, the final RATE_SIZE and sampleAverage are:

**Table 5:** Comparison of Mean Absolute Error (MAE) for heart rate for different values of RATE_ SIZE.

Measurements	RATE_SIZE	MAE
	[–]	[bpm]
20	4	18.5
20	6	4.4
20	8	12.0

• RATE_SIZE = 6,
• sampleAverage = 8.

### 7.3 Validation

One smartwatch was constructed and configured to collect data. It was used by two individuals at different times, resulting in sixty measurements along with their respective surveys (see Table 6).

This smartwatch was engineered using open-source hardware and software, which provides several advantages as well as certain limitations. The advantages of this smartwatch are:

**Table 6:** Dataset of thermal surveys and measurements. The data includes clothing insulation level ($I_{cl}$), metabolic rate ($M_{r}$), location (Loc), thermal sensation vote (TSV), thermal acceptance (TA), wrist skin temperature ($T_{w}$), heart rate ($H_{r}$), age, weight (W), height (H), sex (S), frequency of use of air-conditioned spaces (F), and the individual (I) who completed the survey.

Date	$I_{cl}$	$M_{r}$	Loc	TSV	TA	$T_{w}$	$H_{r}$	Age	W	H	S	F	I
YY-MM-DD HH:MM	[clo]	[met]	[–]	[–]	[–]	[°C]	[bpm]	[year]	[kg]	[m]	[–]	[–]	[–]
2024-07-17 13:32	0.21	1.5	Aula 1	0.0	Yes	34.3	66	28	65	1.70	M	1	1
2024-07-17 14:32	0.21	1.5	Aula 1	0.0	Yes	35.4	77	28	65	1.70	M	1	1
2024-07-17 16:45	0.21	2.0	Aula 1	2.0	No	35.3	85	28	65	1.70	M	1	1
...	...	...	...	...	...	...	...	...	...	...	...	...	...
2024-11-13 08:46	0.48	1.3	Aula 2	0.0	Yes	33.6	62	46	73	1.70	M	1	2
2024-11-14 16:45	0.40	1.3	Aula 2	0.0	Yes	36.9	64	46	73	1.70	M	1	2
2024-11-14 20:45	0.40	1.3	Aula 2	0.0	Yes	35.0	93	46	70	1.70	M	1	2

• Hardware and software are open-source, creating a fully open ecosystem that seamlessly integrates both comfort surveys and physiological measurements within a single wearable.
• Sensors used are inexpensive and readily available.
• All sensors and the screen use I2C, a well-established communication standard.
• The design is tailored to fit into an ecosystem where temperature and relative humidity are measured in spaces used by users.
• Additional I2C sensors for ambient temperature and humidity can be integrated.
• All information is digitized and stored on the IoT platform, enhancing accessibility and remote monitoring.
• The MAE of the skin temperature sensor is 0.3 °C, which is the maximum acceptable by the ISO 80601-2-56:2017 in temperature ranges between 34 °C to 43 °C [21].
• The heart rate sensor achieved an error of 4.4 bpm, which is below the 5 bpm threshold specified by ISO 81060-2:2013 [22].

However, there are limitations to consider:

• The inexpensive nature of the sensors may require calibration.
• The case design can be improved by enhancing aesthetics, comfort, and increasing both shock and water resistance.
• A 240 × 240 capacitive screen may lack the visual clarity and touch sensitivity of more advanced models.
• The ROM memory used of the MCU XIAO ESP32C3 was about 80%, an MCU with more capacity may be needed if the provided code is expanded.

## 8 Conclusion

An open-source IoT smartwatch designed to collect data for thermal comfort studies has been presented. The smartwatch simplifies the collection of thermal comfort surveys and measures skin temperature and heart rate. Surveys are completed through the smartwatch touchscreen, and the device is programmed to prompt the user with a vibration alarm when it is time to answer the survey. During the assignment of the smartwatch, additional user information such as age, weight, height, sex, and frequency of air-conditioned space usage was collected. This data allows for the creation of databases that can be used to develop adaptative and location-specific thermal comfort models. The use of low-cost sensors required calibration. After calibration, the mean absolute error for skin temperature was 0.41 °C, and for the heart rate measurements it was 3.6 bpm. One smartwatch was developed and calibrated. Sixty measurements and their corresponding surveys were collected from two measurement campaigns conducted with different individuals. A Jupyter notebook was shared in the repository to collect user data during their use of the smartwatch. These results demonstrate that the smartwatch is capable to collect data that can be used to develop thermal comfort models, which is not the scope of this paper. The smartwatch does not include ambient temperature and relative humidity measurements, as it is designed for use in a building where such measurements are provided by sensors in each space. However, the smartwatch’s measurement capabilities can be expanded to include ambient temperature and relative humidity. The smartwatch is a valuable tool for researchers seeking to collect data in diverse environments without the need for extensive and controlled measurement campaigns. A fully open ecosystem that seamlessly integrates both comfort surveys and physiological measurements within a single wearable has been developed.

## CRediT authorship contribution statement

**Julio Landa:** Writing – original draft, Visualization, Validation, Software, Methodology, Investigation, Formal analysis, Conceptualization. **Guillermo Barrios:** Writing – original draft, Supervision, Resources, Project administration, Data curation. **Guadalupe Huelsz:** Writing – review & editing, Resources, Methodology.

## Ethics statements

Since our study involves the participation of humans, consent was obtained from the volunteers who used the smartwatch.

## Declaration of competing interest

The authors declare that they have no known competing financial interests or personal relationships that could have appeared to influence the work reported in this paper.
